# Tops and Trends in Iranian Cancer Research: A Bibliometric Analysis

**DOI:** 10.34172/aim.2022.38

**Published:** 2022-04-01

**Authors:** Mozaffar CheshmehSohrabi, Rasoul Shabani, Shiva Shirdavani

**Affiliations:** ^1^Department of knowledge and Information Science, Faculty of Education and Psychology, University of Isfahan, Isfahan, Iran

**Keywords:** Bibliometrics, Cancer, Iran, Keyword analysis, Social network analysis

## Abstract

**Background:**

Detecting the main actors and important topics of Iranian cancer research is essential for Iranian policymakers, clinicians, and researchers. This study was conducted to demonstrate the trends and tops in Iranian cancer research from 1960 to 2018.

**Methods:**

A total of 22,370 Iranian cancer articles in Web of Science (WoS), PubMed, and Scopus, from 1960 through 2018, were extracted and preprocessed based on data mining techniques and analyzed using the collaboration network analysis, keywords analysis, and bibliometrics methods.

**Results:**

The results reveal that, Tehran University of Medical Sciences (11.46%) among organizations, *Asian Pacific Journal of Cancer Prevention* (5%) among journals, Malekzadeh R (1.09%) among authors, and Breast cancer (10.37%) among topics ranked the first. The trend of Iranian cancer research represents three periods: 1) germinating period, from 1970 to 2000, 2) developing period, from 2002 to 2014, and 3) flourishing period, from 2014 to 2018. It is expected that this trend will continue. The results indicate an average 12.8% increase in the logarithm of the count of articles published by Iranian cancer researchers each year. The findings are contextualized with Price’s publications trends principal for determining global and Iranian cancer research publication trends.

**Conclusion:**

The number of research papers published by Iranian researchers on cancer is increasing. In order to maintain the publication growth in this field, greater participation by other Iranian institutions is suggested. Although the quantity and quality of papers are increasing in some topics, certain topics and types of cancers should be still further studied and the Iranian policymakers should be encouraged to invest more in these topics.

## Introduction

 According to the estimates from the World Health Organization (WHO) in 2019, cancer is the first or second leading cause of death before the age of 70 years in 112 of 183 countries and ranks third or fourth in 23 countries.^1^ More than 100 types of cancer have been identified worldwide.^2^ In the past, cancers were regarded as the main cause of mortality,^3^ while now they are the second leading cause of death with 1 in 6 deaths globally, (i.e. an estimated 10 million deaths in 2020). Approximately 70% of deaths caused by cancer occur in low- and middle-income countries.^4^ The most common cancers pertain to the breast, lung, colon and rectum, prostate, skin and stomach, respectively.^4^ It is estimated that by 2035, one quarter of the global population will be directly affected by cancers.^5^ According to WHO, high body mass index, low fruit and vegetable intake, lack of physical activity (sedentarism), and tobacco and alcohol consumption constitute the sources of one third of cancer deaths.^4^ There are also other reasons, like population aging^6^ and changes in reproduction rate.^7^

 Worldwide, cancer cases reached 17.5 million in 2015, with 8.7 million deaths.^6^ In 2020, based on the WHO data, the cancer rate in Australia, New Zealand, Ireland, USA, and Denmark broke the record by more than 350/100 000. Niger, Gambia, Nepal, Congo, Tajikistan, Djibouti, Sudan, Benin, Saudi Arabia, Yemen, and India have, in turn, a record of less than 100/100 000, while Iran has a rate of about 153/100 000.^8^

 In Iran, among the 131 191 new cancer cases in 2020, the five most common cancer types included breast (12.9%), stomach (11.2%), colorectum (9.1%), lung (8%), and prostate (6.8%). The most common cancers among males were those of the stomach (13.6%), prostate (12.6%), lung (10.2%), colorectum (9.7%), and bladder (6.1%), while for females, breast (28.1%), colorectum (8.4%), stomach (8.4%), lung (5.4%), and thyroid (5.2%) cancers were the most prevalent.^9^ In contrast, stomach cancer (16.4%) was the leading cause of cancer deaths^1^ followed by lung (11.5%), liver (6.7%), brain and central nervous system (6.7%), and breast (6.1%) cancers.^9^

 Based on cancer entries information in Medical Subject Headings (MeSH) among all diseases, cancer is one of the most active and dynamic areas focused on by researchers with emerging fronts and topics. During the last 50 years, the volume of scientific documents on oncology has increased rapidly.^10^ Publications on the 26 cancers types amounted to 8.19% in PubMed, and 8.04% in Web of Science (WoS).^11^ Based on the Scopus data, from 3 047 844 indexed documents until 30 December 2019 on all topics of cancer, 22 696 records (0.744%) belong to Iran. Based on the WoS data, from 2 354 917 documents, Iran held 18 955 (0.804%) of the records. In the same period, the US held 827 457 records, (35.13%), the top rank.

 The scientific collaboration of the authors of the documents is an important issue that is analyzed based on the three indicators of degree, closeness, and betweenness centralities. As to degree centrality, actors who have more ties to other actors may be in high positions due to which, they may have alternative manners to justify their scientific needs, thus, have less dependency on others if any. They are often the third parties and dealmakers in exchanges among others and are able to benefit from this brokerage.^12^ Therefore, in a co-authorship network, the degree centrality of each author indicates the times he/she has co-authored with those present in the network. As to closeness centrality, according to Hanneman and Riddle “*closeness centrality approaches emphasize on the distance of an actor to all others in the network by focusing on the geodesic distance from each actor to all others*”.^12^ As to betweenness, according to Hanneman and Riddle “*views an actor as being in a favored position to the extent that the actor falls on the geodesic paths between other pairs of actors in the network. That is, the more people depend on me to make connections with other people, the more power I have”*.^12^ According to Abbasi et al, the nodes with high betweenness centrality are highly contributive in the network connection. They have a core position in the network and can promote information flow in the network.^13^ One of the important issues in the analysis of these indicators is the detection of *“elite groups*”. According to Tsvetovat and Kouznetsov “*every network has a certain elite group of users that will be noticeable in the same way—often, two out of three, or all three centrality metrics will land a person in the top ten. If the target use of the analysis is for directed advertising, information operations, or intelligence collection (whether for business or government), these elite groups form a perfect target”*.^14^

 Bibliometrics is *the application of mathematics and statistical methods to books and other media of communication.*^15^ Its history goes back to Campbell’s research on subject scattering in publications in 1896.^16^ For the first time in 1977, the issue of using bibliometrics in cancer research was raised by Wagner and Sandor, although the history of cancer literature analysis dates back to decades before this and the works of Wolff- Tehhoine in 1966. Due to the importance of the cancer and related fields, this field has integrated bibliometricians during recent years. A survey run on the three databases of the WoS, PubMed and Scopus indicates that 150 bibliometric analyses were run in this field by Sandor et al, Grossi et al, Micheli et al, Thonon et al, Wang et al, Ruiz-Coronel et al, and others.^17-22^ The analysis run on these 150 researches reveals that the highest frequency is in 2016 (n = 17), 2017 (n = 27), and 2018 (n = 18). The three subject categories of 1) research productivity, 2) citation analysis, and 3) emerging topics or technologies are ranked at the top, with 96, 23, and 7 studies, respectively. Among cancer type, the most bibliometric analyses are run on breast, lung, and oral cancers with 19, 12 and 7 researches, respectively. WoS is the source of extracted data in 76 bibliometric studies, followed by PubMed and Scopus with 26 and 20, respectively. Based on the results of these 150 studies, USA is at the top with 36 studies.

 Three types of the data: (1) concerning cancer and its incidence and mortality in the world and Iran, (2) the data related to cancer research, and (3) regarding growth and analysis of cancer literature are of concern. These data reveal that there is a significant correlation among cancer incidence, treatment, mortality rate and cancer research rate.

 The subject of bibliometric analysis of Iranian cancer research and related topics have made some researchers^23-28^ interested in the following three emerging categories:

1. The Iranian cancer research, where Foroughi et alreviewed the Iranian research on the oncology field during 1995–2015 based on the WoS and Scopus databases and reported that Iran had 2865 articles on cancer, with an H-index score of 52 and *Asian Pacific Journal of Cancer Prevention* was the top journal where the Iranian articles on cancer were published.^23^
2. The specific cancer type research in Iran, where Ghojazadeh et alanalyzed the gastric cancer field studies based on the Medline data during 2000–2011, and found that the mean cooperation coefficient among the researchers was 6.14 ± 3.29 person/article. Tehran University of Medical Sciences and Mohammadreza Zali were at the top in terms of publishing scientific articles.^24^ Zand Vakili and Rasolabadi assessed Iran’s publication performance in ovarian cancer during 1996–2015 by analyzing 386 articles and found that the Iranian ovarian cancer research increased from 25 articles during 1996–2005 to 360 articles during 2006–2015. The average citation per article for Iran’s publication outputs was 5.72.^25^ More recently, Zarei et al analyzed the Iranian researchers’ performance on breast cancer in Scopus database during 1991–2015, and found that the count of the domestic articles on breast cancer and the count of citations therein, is on the increase. Iran has 2399 published articles with an average of 95.96 per annum and an average 6.49 citations/article.^26^ Biglu and Tabatabaei evaluated gastrointestinal cancers research in Medline and Scopus databases during 2002–2012, and extracted 2294 articles in terms of GI cancer written by Iranian researchers in Scopus and Medline with Tehran University of Medical Sciences as the organization and *Asian Pacific Journal of Cancer Prevention* as the publication at the top.^27^
3. The co-authorship network where Osareh et almapped the Iranian cancer researchers’ co-authorship network based on WoS data during 2000–2013, and found a clustering coefficient of 0.57% and a density of 10%. Oncology, occupational health, digestion and internal medicine researchers had the highest collaboration levels in this network. Mohagheghi, Mahmoudi, and Malekzadeh were identified as the most influential Iranian researchers on cancer.^28^

The available bibliometric contributions in Iranian cancer research is somewhat restricted due to: (1) low volume of analyzed data, 92) one or two database as to the count of assessed databases therein, (3) the count of verified topics where one specific cancer or one theme like co-authorship network is assessed in most studies, and (4) the specific verified time period of 10 years. Of course, each one of these bibliometric analyses provided a clear *snapshot* of one aspect of the overall cancer issue in Iran. It is necessary to conduct a study that would provide a comprehensive perspective on cancer research in Iran from the beginning up to the date. In addition, previous research did not examine the status of cancer publications with the principle of the Price publication trends. In this study, the results of the research are conceptualized with this principle.

 In this context, the objective of this article is to realize and demonstrate the Iranian cancer research field from 1960 to 2018. The issues of concern here consist of: (1) the Iranian cancer research trends, and (2) the tops in Iranian cancer research.

## Material and Methods

 The method adopted in this study was descriptive and the section regarding the identification of trends and topics and the co-authoring network was exploratory. The statistical population of the study consisted of 22 370 Iranian cancer articles available in the three WoS, PubMed and Scopus databases. Because the whole population was analyzed, no sampling was deemed necessary, and consequently, the census method was applied.

###  Data Source

 The three databases of WoS, PubMed and Scopus were selected, because none of them was comprehensive on its own. Data collection, preprocessing and analysis were done in 2019.

###  Search Strategy

 After selecting the subject databases, the following steps were followed by the research team: first, the cancer keyword was searched in the MeSH. Among the terms that appear below the entry of neoplasms, the terms including cancer, malignancy, neoplasia, tumor, tumour and carcinoma were chosen, and next, the search formula for each database was devised.

###  Data Preprocessing 

**Figure 1:**
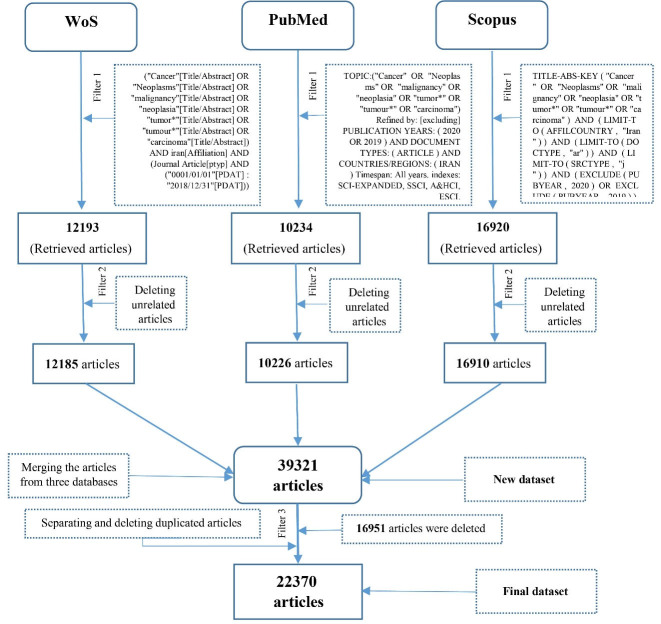
Stages of Dataset Formation.

 Data preprocessing was run based on data mining techniques (data integration, cleaning, transformation and reduction). Data preparation was made in the following four steps:

1. The data from the subject databases were merged and a new dataset was generated with 39 347 articles.
2. Twenty-six unrelated and 16 951 duplicate records were deleted leaving a total of 22 370 articles. Figure 1 shows the steps and filters that were applied. The coverage and overlap of these 22 370 articles in all three databases of WoS, PubMed, and Scopus are specified in Table 1.
3. The fields required for data analysis, (i.e. author, title, source title, affiliation, date, and keyword) were selected and the rest were deleted.
4. Cleaning the data and information of the selected fields began, in which the selected fields were stored in separate files and converted into the text format. These files were then converted into a format that could be identifiable by the PriMap software. The authors’ names were cleaned. Because the name of some authors were sometimes written in full or abbreviated, an attempt was made to have only the full name. The journal titles were reviewed and cleaned, and matched with the Scimago and the JCR journals. The organizational affiliation of the authors was assessed. To match the names of institutions and universities, the names of each were searched in Google. After finding the official site of the institution or university, their titles were adjusted according to the official titles in the websites. In this context, all Islamic Azad University (IAU) branches were omitted and IAU would represent all. All research institutes, research centers, departments, hospitals and laboratories under the supervision of a university or scientific institute were renamed into the name of the principal institution. The keywords were reviewed and cleaned, where keywords containing meaningless signs, numbers, letters, words, or symbols were removed.

**Table 1:** Status of 22 370 Articles Based on the Amount of Coverage and Overlap at WoS, PubMed and Scopus

**Database**	**Count of Article**	**%**
Scopus	5978	26.7
WoS	2902	13
PubMed	2361	10.6
WoS & Scopus	3272	14.6
WoS & PubMed	271	1.2
Scopus & PubMed	1854	8.3
WoS, PubMed & Scopus	5732	25.6
Total	22 370	100

###  Data Analysis 

 Excel, Netdraw,^29^ UCINET,^30^ and PreMap^31^ software packages were applied for data analysis, which was based on collaboration network analysis, keyword analysis, and bibliometric methods.

 In order to draw the authors’ collaboration network, because the count of authors in the first group was more than 1700 and the network was not clear, the minimum frequency was ≥ 50, and finally the first 94 authors’ scientific collaboration network was drawn. The co-authorship matrix of the authors was prepared, and the matrix was transferred to UCINET software, and subsequently, the authors’ correlation matrix was made and stored. In Netdraw software, the collaborative network was visualized and mapped. UCINET software was applied to calculate the degree, closeness, and betweenness centralities.

## Results

###  Publication Trend Analysis 

**Figure 2:**
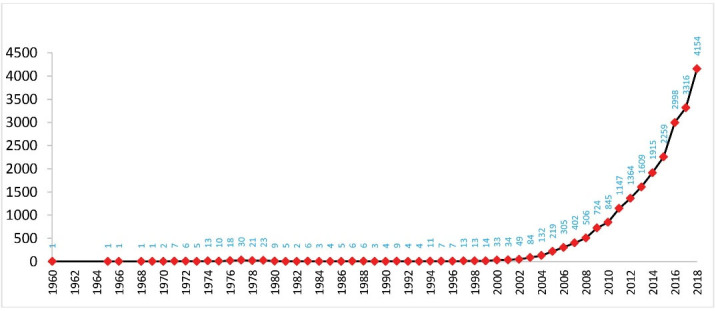
Publication Years of Iranian Cancer Articles Based on WoS, PubMed, and Scopus.

 The bibliographic data of 22 370 articles was retrieved. In the 1960s, a total of five articles were published. From 1970 to 2000, the publication count fluctuated between 2 and 33 articles /year. As observed in Figure 2, the count of Iranian articles on cancer, beginning with the first in 1960 to 2000, followed an almost steady growth trend, while, beginning in 2002, it grew from 34 to 4154 articles in 2018. For a five-year period, 2014 to 2018, 14 642 articles were published, i.e. 65.45%. The highest output pertained to 2018 at 4154 articles (18.57%).

**Figure 3:**
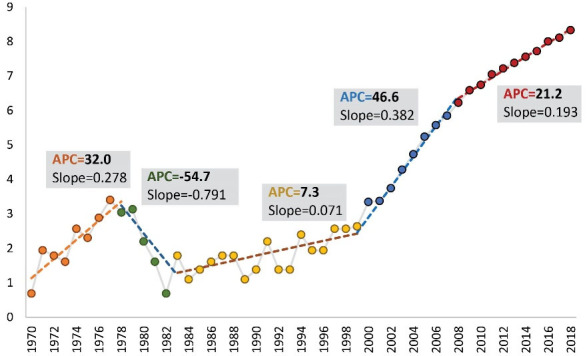
Fitted Joinpoint Regression Model.

 Furthermore, the average annual percent change (AAPC) during different time periods was provided using the joinpoint regression model which was fitted to the logarithm of the count of articles published by Iranian cancer researchers. The number of joinpoints in this model was chosen according to the Akaike information criterion (AIC). The fitted regression model is illustrated in Figure 3. The vertical axis indicates the logarithm of the count of articles published by Iranian cancer researchers.

 Taking advantage of the fitted joinpoint regression model, the authors employed the annual percent change (APC) to estimate the average of change in the logarithm of Iranian cancer research. As such, *logY = a + bx* was the fitted regression model for a specific segment among five segments in Figure 3, where x is the year and b denotes the slope parameter. The APC index for this segment equals to (*exp*(b)-1) × 100 which indicates the average amount of change in logY per year in that segment. The value of APC for each segment is demonstrated in Figure 3. The estimation of slope parameter, its standard error, APC, and the coefficient of determination (R-squared) for every five segments can be found in Table 2.

**Table 2:** Summary Statistics of the Fitted Joinpoint Regression Model

**Window**	**Period**		**Average of Logarithm of #Articles**	**R-Squared**	**Slope**	**SE**	**APC**
1	1970	1978	2.249	0.825	0.278	0.048	32.008
2	1979	1982	1.909	0.993	-0.791	0.049	-54.683
3	1983	1999	1.823	0.493	0.071	0.018	7.316
4	2000	2008	4.840	0.982	0.382	0.020	46.584
5	2009	2018	7.469	0.995	0.193	0.005	21.244

APC, annual percent change; SE, standard error.

 A weighted average of APC for different segments can be computed to describe the AAPC for the whole period of 1970–2018. AAPC equals to $(\exp\left(\sum_{j-1}^{k}w_{j}b_{j}\right)-1)\times 100$ , where *w*_j_ and *b*_j_ are the width of the segment *j* and the estimated slope parameter, respectively. Here, AAPC = 12.80 which indicates an average of 12.8% increase in the logarithm of the count of articles published by Iranian cancer researchers each year. A 95% parametric confidence interval for AAPC is also computed as L = 6.68 and U = 210.13, which indicates a minimum of 6.68% increase in the logarithm of the count of articles published by Iranian cancer researchers in average per year. The given parametric confidence interval holds regarding the asymptotic distribution of the sample mean according to variants of the well-known central limit theorem in statistics.

###  Institutional Analysis

 A total of 1732 institutions contributed to the publication of 22 370 articles on Iranian cancer research. The contribution of the institutions with more than 1% is shown in Table 3. The status of 21 812 remaining contributions is shown in Table 4.

**Table 3:** Contribution of the Institutions with More Than 1% Contribution

**Rank**	**Institution**	**Contribution Count**	**%**	**City**
1	Tehran University of Medical Sciences	5603	11.46	Tehran
2	Shahid Beheshti University of Medical Sciences	3323	6.79	Tehran
3	Islamic Azad University	2538	5.19	—
4	Shiraz University of Medical Sciences	1954	3.99	Shiraz
5	Mashhad University of Medical Sciences	1931	3.95	Mashhad
6	Tabriz University of Medical Sciences	1849	3.78	Tabriz
7	Isfahan University of Medical Sciences	1677	3.43	Isfahan
8	Iran University of Medical Sciences	1444	2.95	Tehran
9	Tarbiat Modares University	1385	2.83	Tehran
10	University of Tehran	1170	2.39	Tehran
11	Pasteur Institute of Iran	738	1.51	Tehran
12	Mazandaran University of Medical Sciences	673	1.38	Sari
13	Kermanshah University of Medical Sciences	608	1.24	Kermanshah
14	Academic Center for Education, Culture and Research	601	1.23	Tehran
15	Ahvaz Jundishapur University of Medical Sciences	551	1.13	Ahvaz
16	Baqiyatallah University of Medical Sciences	543	1.11	Tehran
17	Kerman University of Medical Sciences	503	1.03	Kerman
Total		27091	55.39	

**Table 4:** Contributions of Other Institutions

**Category**	**Institution Count**	**Times Contributed**	**Total Contribution**	**%**
1	615	1	615	1.26
2	232	2	464	0.95
3	139	3	417	0.85
4	94	4	376	0.77
5	60	5	300	0.61
6	208	6–10	1605	3.28
7	188	11–20	2757	5.64
8	102	21–50	3153	6.45
9	31	51–100	2253	4.61
10	36	101–300	6213	12.71
11	10	301–500	3659	7.48
Total	1715	—	21 812	44.61

###  Author Analysis

 In total, 61 984 authors have written 22 370 articles, and the top 10 are listed in Table 5. The 10 top authors have published 1590 articles (7.1%) out of 22 370 (100%).

**Table 5:** The Top 10 Iranian Cancer Authors According to WoS, PubMed and Scopus

**Rank**	**Author**	**Affiliations**	**Count of Articles**	**%**
1	Malekzadeh R	Tehran University of Medical Sciences	243	1.09
2	Zali MR	Shahid Beheshti University of Medical Sciences	213	0.95
3	Ghaderi A	Shiraz University of Medical Sciences	172	0.77
4	Baradaran B	Tabriz University of Medical Sciences	167	0.75
5	Ghavamzadeh A	Tehran University of Medical Sciences	166	0.74
6	Akbari ME	Shahid Beheshti University of Medical Sciences	148	0.66
7	Ramezani M	Mashhad University of Medical Sciences	123	0.55
8	Zarghami N	Tabriz University of Medical Sciences	122	0.54
9	Sahebkar AH	Mashhad University of Medical Sciences	121	0.54
10	Avan A	Mashhad University of Medical Sciences	115	0.51

###  Collaboration Network Analysis 

 Every author’s performance was assessed based on three indicators of degree, closeness, and betweenness centralities, and the top 10 are listed in Table 6.

**Table 6:** Ten Iranian Authors with the Highest Degree, Closeness and Betweenness Centralities

**Rank**	**Degree**		**Closeness**		**Betweenness**	
	**Author**	**Value**	**Author**	**Value**	**Author**	**Value**
1	Malekzadeh R	80	Heshmat R	11633	Yazdani Y	11287.95
2	Sahebkar AH	27	Moradi A	11657	Sahebkar A.H	9269.23
3	Ghavamzadeh A	16	Yazdani Y	11660	Ghanbari R	8152.56
4	Zali MR	15	Kelishadi R	11676	Heshmat R	7882.71
5	Baradaran B	9.7	Ghanbari R	11683	Mahdavi M	7181.14
6	Akbari ME	9.5	Nahvijou A	11700	Vasei M	6946.60
7	Ghaderi A	8.5	Mesdaghinia A	11708	Mahmoudi M	6860.86
8	Avan A	8.1	Nowroozi MR	11710	Kelishadi R	6835.13
9	Zarghami N	5	Keshtkar AA	11715	Moradi A	6832.32
10	Ramezani M	4	Parsian H	11720	Hadjati J	6455.71

 As observed in Table 6, these authors are of proper status in the network, considered to be the network leaders based on the shortest paths among other authors, and can control the information flow on the network.

 Here, the degree centrality refers to the total count of co-authorships of an author. The results obtained from the calculation of the degree centrality show that Malekzadeh with 80 degree centrality ranked first followed by Sahebkar, and Ghavamzadeh. This shows that they are the most active and visible researchers and have more influence in the co-authorship network of the field of Iranian cancer. In terms of closeness centrality, Heshmat is on top followed by Moradi and Yazdani. These authors are the closest or more central authors of the network of the field of Iranian cancer. The most influential author in the co-authorship network of the field of Iranian cancer is Yazdani given that he and other authors with high betweenness centrality play the role of a connector of nodes/clusters and controller of the flow of information in the network. Moreover, the examination of the top authors in the co-authorship network of the field of Iranian cancer based on three measures of centrality (i.e., degree, closeness, and betweenness) reveals that some researchers are in two of these three measures. In network analysis, actors who fall into two or all three measures are called the “elite group” of the network.^14^ With this interpretation, the elite group of the co-authorship network in the Iranian cancer field is composed of Yazdani, Heshmat, Sahebkar, Moradi, Ghanbari, and Kelishadi (Table 6, are colored).

 To analyze the correlation among Iranian authors in this respect, their collaboration was drawn by applying UCINET and Netdraw. Each author was considered as a node, and his/her scientific collaboration was determined in co-authorship context based on the network ties.

###  Degree Centrality 

**Figure 4:**
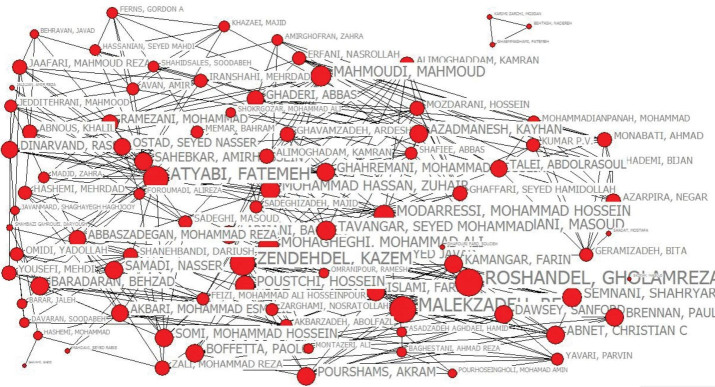
A perspective of the Scientific Collaboration Network of Iranian Authors on Cancer Based on Degree Centrality Index.

 Each node size indicates the times one has co-authored. This network covers 89 nodes (authors) consisting of two clusters (Figure 4), where the main cluster contains 86 nodes and the second 3. An assessment run on the degree centrality revealed that Malekzadeh R has contributed the most to the publication of articles in this field in Iran, followed by Sabekkar, Ghavamzadeh, and Zali (Table 3).

###  Closeness Centrality

**Figure 5:**
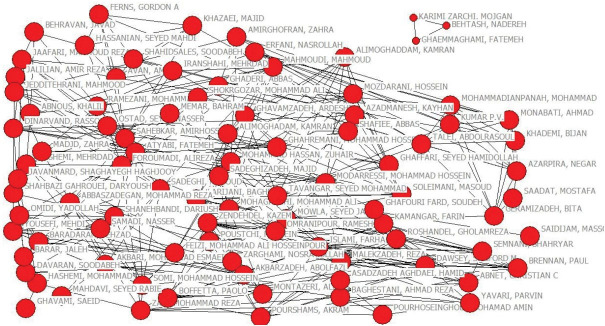
A Perspective of Scientific Collaboration Network of Iranian Authors on Cancer Based on Closeness Centrality Index.

 The closeness centrality network includes 92 nodes in two clusters: the main cluster with 89 nodes and the second with 3 (Figure 5).

###  Betweenness Centrality

**Figure 6:**
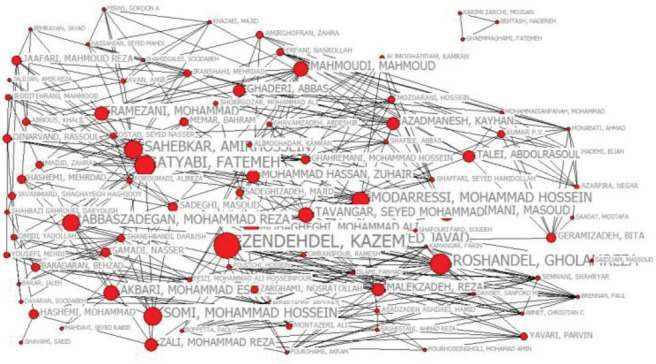
A Perspective of the Scientific Collaboration Network of Iranian Cancer Authors Based on Betweenness Centrality Index.

 The betweenness centrality index of the author is measured based on his/her status in the network, that is, the more the betweenness centrality, the more his/her centrality with respect to node count. The diameter of the nodes (circles) indicates the measure of betweenness centrality, that is, larger nodes have more betweenness centrality than smaller nodes (Figure 6) This network consist of 89 nodes in two clusters: the first with 86 nodes and the second with 3.

###  Journal Analysis 

 A total of 22 370 articles were published in 3645 journals. The top 18 journals published 3942 (17.62%) articles.

*The Asian Pacific Journal of Cancer Prevention* (n = 1126, 5%) ranked the first. Among the top 18 journals, 12 were from Iran, three from the United States, two from India and one from Thailand. The impact factors of 10 journals were above 1.00. Six of these journals were specific to cancer studies (Table 7).

**Table 7:** Top 18 Journals with Most Iranian Cancer Researchers’ Articles in WoS, PubMed, and Scopus

**Rank**	**Journal Title**	**Journal IF (2018)**	**Country**	**Frequency**	**%**
1	Asian Pacific Journal of Cancer Prevention	2.51	Thailand	1126	5.03
2	International Journal of Cancer Management	0.79	Iran	295	1.31
3	Archives of Iranian Medicine	1.14	Iran	269	1.20
4	Journal of Isfahan Medical School	0.18	Iran	264	1.18
5	Tehran University Medical Journal	0.26	Iran	201	0.9
6	Acta Medica Iranica	0.88	Iran	187	0.83
7	Journal of Mazandaran University of Medical Sciences	0.45	Iran	177	0.79
8	Iranian Journal of Cancer Prevention	—	Iran	173	0.77
9	Iranian Journal of Pharmaceutical Research	1.18	Iran	154	0.69
10	Iranian Red Crescent Medical Journal	0.64	Iran	154	0.69
11	Journal of Research In Medical Sciences	1.46	Iran	151	0.67
12	Journal of Cancer Research and Therapeutics	1.39	India	127	0.57
13	International Journal of Hematology Oncology and Stem Cell Research	—	Iran	124	0.55
14	Iranian Journal of Basic Medical Sciences (IJBMS)	1.85	Iran	120	0.53
15	Tumor Biology	3.65	USA	116	0.51
16	PLoS One	2.77	USA	106	0.47
17	Iranian Journal of Medical Sciences	1.15	Iran	101	0.45
18	Artificial Cells Nanomedicine and Biotechnology	4.46	USA	97	0.43

###  Keyword Analysis

 Analysis run by applying keywords is contributive in identifying the most important topics in any field. The keywords of all 22 370 articles were analyzed using Excel, Netdraw and UCINET. In total, 111 103 keywords were assigned to 22 370 articles, of which 83 050 were duplicates (reset 28 053). Of the 28 053 keywords, 18,157 keywords (64.72%) were applied only once, followed by 3906, twice and 1722, three times. About 79% (n = 22 063) of keywords were applied once or twice in 22 370 articles, 90% (n = 25 326) of keywords were applied one to five times. Among 28 053 keywords, 751 had a frequency greater than 20.

 According to Bradford’s law, 249 core topics were identified. *Breast cancer* (n = 2319, in 10.37% articles) ranked first, followed by *apoptosis* (n = 1092, 4.88%), and *in vitro* (n = 791, 3.53%). The first 25 core topics are shown in detail in Table 8.

**Table 8:** First 25 Core Topics with the Most Count

**Rank**	**Topic**	**Frequency**	**%**
1	Breast cancer	2319	10.37
2	Apoptosis	1092	4.88
3	In vitro	791	3.53
4	Colorectal cancer	656	2.93
5	Gastric cancer	621	2.77
6	Nanoparticle	587	2.62
7	Chemotherapy	577	2.57
8	Prostate cancer	536	2.39
9	Protein	472	2.11
10	Lung cancer	462	2.06
11	Cancer cells	454	2.03
12	Metastasis	441	1.97
13	Cytotoxicity	428	1.91
14	Gene	408	1.82
15	Diagnosis	373	1.66
16	Epidemiology	371	1.65
17	MicroRNA	360	1.61
18	Risk factor	359	1.6
19	Polymorphism	346	1.55
20	Squamous cell carcinoma	338	1.51
21	Colon Cancer	334	1.49
22	Oxidative stress	331	1.48
23	Gene expression	320	1.43
24	Vivo	309	1.38
25	Prevalence	309	1.38

 In terms of cancer types, three clusters were identified including (1) the cancers that have received a lot of attention such as breast cancer (n = 2319), colorectal cancer (n = 565), gastric cancer (n = 621), prostate cancer (n = 536), lung cancer (n = 621), colon cancer (n = 334), esophageal cancer (n = 282), cervical cancer (n = 266), and ovarian cancer (n = 216), (2) the cancers that have received relatively little attention such as thyroid cancer (n = 141), skin cancer (n = 111), neck cancer (n = 104), and pancreas cancer (n = 99), laryngeal cancer (n = 24), and (3) the cancers that have been addressed in a maximum of ten articles such as kidney cancer (n = 8), blood cancer (n = 7), tongue cancer (n = 4), lip cancer (n = 4), vulvar cancer n = 3), gallbladder cancer (n = 3), penile cancer (n = 2), soft tissue cancer (n = 2), pelvic cancer (n = 2), mesothelioma cancer (n = 1), lymphoma cancer (n = 1) and eye cancer (n = 1).

## Discussion

 As Price stated, the publication trend has doubled within a period of 10 to 15 years,^32^ while the findings of the present study revealed that this doubling in volume in Iran began from 2000 in three-year intervals. The average annual growth of Iranian cancer articles from 2000 to 2018 was 37.48%. The growth rate of cancer articles at the global scale is in line with Price’s prediction in 1963 and has doubled every ten years from 1990 to 2019, but the growth rate of Iranian cancer articles is at odds with Price’s and has doubled every three years. An annual average of 22.68% for the 2010–2019 period based on the WoS data confirms the Chawla report, indicating a growth of 22% per annum between 2004 and 2014 based on Scopus data where the Iranian authors have the fastest-growing presence in the overall scientific and engineering literature^33^; it is probably related to the major changes in Iran’s educational and research policies in the last two decades, and also to the rapid development of ICT affordances leading to more researchers’ access to scientific resources. Kshitij et alreported an average annual growth rate of about 18% for Indian cancer research from the WoS database during 2000–2011.^34^

 Malekzadeh R was the most prolific author with 243 articles and more than one percent contribution (1.09%) in the publication of Iranian cancer articles. In the co-authorship network of Iranian cancer articles, Yazdani Y, Sahebkar AH, and Ghanbari R were the top authors based on betweenness centrality. These authors with high betweenness centrality are highly contributive in the network connection. As such, they have a core position in the network and can promote information flow in the network.^13^ Moreover, the degree centrality of each author indicates the times he/she has co-authored with those present in the network. Likewise, Malekzadeh R had a high degree centrality among Iranian cancer researchers. It is important to note that Yazdani, Heshmat, Sahebkar, Moradi, Ghanbari, and Kelishadi fell into two of three measures and constituted the “elite group” of the co-authorship network in the Iranian cancer field.

 In terms of institutions, Tehran University of Medical Sciences was the leader in publishing scientific articles in this field. In term of journals, a significant portion of Iranian cancer articles were published in the renowned journals of *Tumor Biology* and *Asian Pacific Journal of Cancer Prevention*.

 Totally, 249 core topics based on Bradford’s law were identified in the present study. The topics such as breast cancer, apoptosis, *in vitro*, colorectal cancer, gastric cancer, nanoparticle, chemotherapy, prostate cancer, protein, lung cancer, cancer cells, metastasis, cytotoxicity, and gene were on top and each was covered in more than 400 articles. Among the first 50 core topics, there were 12 types of cancers including breast, colorectal, gastric, prostate, lung, colon, esophageal, cervical, liver, ovarian, leukemia, and bladder. This finding reveals that Iranian cancer researchers have paid much attention to these issues. On the contrary, the studies run by the Iranian researchers on pancreas, kidney, blood, laryngeal, tongue, lip, vulvar, gallbladder, penile, pelvic, soft tissue, mesothelioma, lymphoma, eye cancers were very few. These findings are consistent with those of Su et alwho reported that some of these cancers are under-researched.^35^

 In conclusion,based on the findings, the number of Iranian research papers on cancer shows an increasing trend representing three periods: (1) *germinating period* from 1970 to 2000, 92) *developing period* from 2002 to 2014, and (3) *flourishing period* from 2014 to 2018. Moreover, the results indicate an average of 12.8% increase in the logarithm of the rate of articles published by the Iranian cancer researchers each year. More than 55% of contributions in the Iranian cancer papers belong to seventeen institutions. In order to maintain publication growth in this field, greater participation by other Iranian institutions is suggested. Although the quantity and quality of papers are increasing in topics such as breast cancer, apoptosis, *in vitro*, colorectal cancer, gastric cancer, nanoparticle, chemotherapy, prostate cancer, protein, and lung cancer, further studies are needed for certain topics and types of cancers, like skin, neck, pancreas, kidney, blood, laryngeal, tongue, lip, vulvar, gallbladder, penile, pelvic, soft tissue, mesothelioma, lymphoma, and eye, with more investment of the Iranian policymakers in these topics.
